# The σ Subunit-Remodeling Factors: An Emerging Paradigms of Transcription Regulation

**DOI:** 10.3389/fmicb.2020.01798

**Published:** 2020-07-29

**Authors:** Rishi Kishore Vishwakarma, Konstantin Brodolin

**Affiliations:** Institut de Recherche en Infectiologie de Montpellier, CNRS, Université de Montpellier, Montpellier, France

**Keywords:** RNAP-binding transcriptional regulators, sigma subunit conformational dynamics, promoter specificity, RbpA, Tuberculosis

## Abstract

Transcription initiation is a key checkpoint and highly regulated step of gene expression. The sigma (σ) subunit of RNA polymerase (RNAP) controls all transcription initiation steps, from recognition of the −10/−35 promoter elements, upon formation of the closed promoter complex (RPc), to stabilization of the open promoter complex (RPo) and stimulation of the primary steps in RNA synthesis. The canonical mechanism to regulate σ activity upon transcription initiation relies on activators that recognize specific DNA motifs and recruit RNAP to promoters. This mini-review describes an emerging group of transcriptional regulators that form a complex with σ or/and RNAP prior to promoter binding, remodel the σ subunit conformation, and thus modify RNAP activity. Such strategy is widely used by bacteriophages to appropriate the host RNAP. Recent findings on RNAP-binding protein A (RbpA) from *Mycobacterium tuberculosis* and Crl from *Escherichia coli* suggest that activator-driven changes in σ conformation can be a widespread regulatory mechanism in bacteria.

## Introduction

**FIGURE 1:**
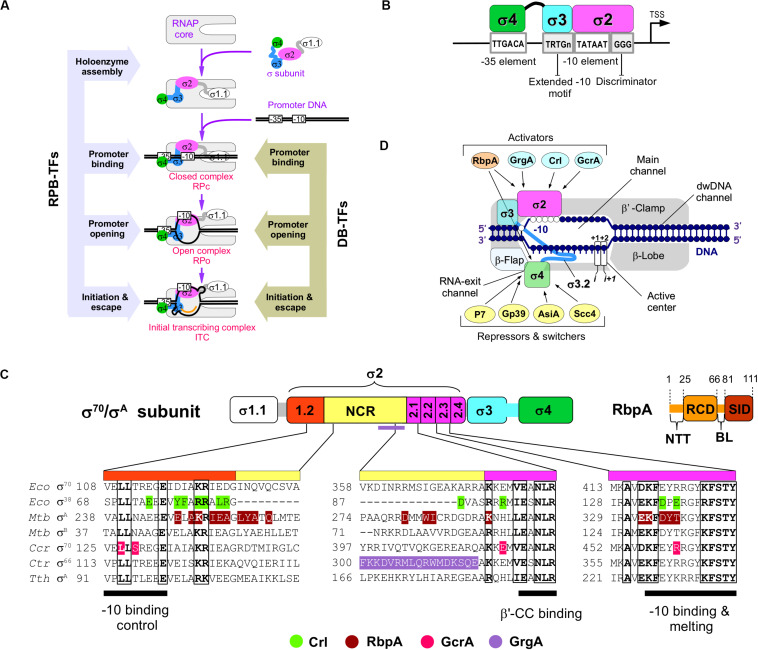
The RNAP-binding σ-regulators and their interaction with RNAP. **(A)** Scheme of the main steps in transcription initiation. The steps regulated by RNAP-binding transcription factors (RPB-TFs) and DNA-binding transcription factors (DB-TFs) are indicated. **(B)** Basal promoter architecture (first described in *E. coli*) and interaction of its key elements with σ-domains. **(C)** Domain organization of the principal σ subunits and RbpA. NCR – non-conserved region, NTT – N-terminal tail, RCD – RbpA core domain, BL – basic linker, SID – σ-interacting domain. Alignment of the σ subunits from *E. coli* (*Eco*), *M. tuberculosis* (*Mtb*), *C. crescentus* (*Ccr*), *C. trachomatis* (*Ctr*), and *T. thermophilus* (*Tth*). Amino acid residues implicated in contacts with activators (bottom) are shown in color. **(D)** Schematic presentation of the RNAP holoenzyme structure with the binding sites for the activators and repressors targeting domains σ2 and σ4, respectively.

Transcription initiation starts with the assembly of the active RNA polymerase (RNAP) holoenzyme from the catalytic core (subunits 2α, β, β′, ω) and the promoter–specificity subunit sigma (σ). The RNAP holoenzyme binds to promoter DNA, and forms a closed promoter complex (RPc) that isomerizes into the open promoter complex (RPo) through several intermediates (Buc and McClure, 1985; Saecker et al., 2011; Boyaci et al., 2019). Upon isomerization, RNAP melts ∼13 bp of DNA duplex between the promoter positions –11 to +2 that encompass the transcription start site (Figure 1A). Recognition of the −10 (*Escherichia coli* consensus motif T_–12_A_–11_T_–10_A_–9_A_–8_T_–7_) and −35 elements (*E. coli* consensus motif T_–35_T_–34_G_–33_A_–32_C_–31_A_–30_) of the promoter (Figure 1B) and promoter DNA melting depend on the σ subunit (Feklistov et al., 2014; Zuo and Steitz, 2015). All bacteria have at least one principal σ subunit [group 1: σ^70^ in *E. coli* and σ^A^ in other species (Gruber and Gross, 2003)] that ensures transcription of most genes [e.g., at least 70% in *Mycobacterium tuberculosis* (*Mtb*)] (Cortes et al., 2014). Alternative σ subunits (groups 2–4) control transcription of specialized sets of genes upon stress response, starvation, and stationary growth (Österberg et al., 2011). Compared with group 1 σ subunits, the group 2 stress-response/stationary phase σ subunits (*E. coli* σ^S^ and *Mtb* σ^B^) lack the N-terminal variable domain σ1.1 and have a shorter non-conserved region (NCR) located in domain σ2 (Figure 1C). RNAPs harboring group 1 and group 2 σ subunits can transcribe the same promoter sets (Rodrigue et al., 2006; Hu et al., 2014). The σ2 domain harbors the highly conserved regions 1.2, 2.1, 2.2, 2.3, and 2.4 that are essential for binding to RNAP β′ clamp, for recognition of the−10 element, and for melting of promoter DNA (Feklistov and Darst, 2011; Zhang et al., 2012). About 73% of the σ2 contact surface with ssDNA of the −10 element is formed by region 2.3 residues. In addition, residues in region 1.2 contact with the T_–7_ base of the −10 element (Feklistov and Darst, 2011) and control recognition of the −10 element allosterically (Zenkin et al., 2007; Morichaud et al., 2016). The σ^70^ NCR interacts with promoter DNA at positions –16/–17 (R157^Eco^) and is implicated in DNA unwinding (Narayanan et al., 2018). The σ^70^ NCR/β′ interaction facilitates promoter escape (Leibman and Hochschild, 2007). Domain σ4 interacts with the β-flap domain of core RNAP and harbors a helix-turn-helix DNA binding domain that recognizes the −35 motif. The σ3 and σ4 subunits are connected by a weakly structured linker (region 3.2) that fills the RNA exit channel and is ejected upon the initial RNA synthesis (Zhang et al., 2012; Li et al., 2020). Most bacterial promoters recognized by group 1 and 2 σ subunits belong to the −10/−35 class and contain the −10 and also the −35 elements. The extended −10 class of promoters (∼20% in *E. coli*) contains the extended −10 motif (T_–17_R_–16_T_–15_G_–14_; R = purine) that is located one base upstream of the −10 element (Keilty and Rosenberg, 1987; Burr et al., 2000; Mitchell et al., 2003) and interacts with the σ3 domain (Barne et al., 1997) (Figure 1B). It has been shown that the extended −10 motif bypasses the requirement of the σ4/−35 element interaction (Kumar et al., 1993). However, σ4 *per se* is essential for transcription initiation by σ^B^-*Mtb*RNAP at the extended −10 promoters (Perumal et al., 2018).

As a general rule, the principal σ subunit, the cellular concentration of which exceeds that of core RNAP (Gaal et al., 2006) should recognize and bind to promoter DNA only in the context of RNAP holoenzyme. Free σ should be devoid of DNA binding activity that might inhibit transcription. Data from structural and biophysical studies suggest that free group 1 and 2 σ subunits adopt a “closed” inactive conformation in which the spatial arrangement of domains σ2 and σ4 is incompatible with promoter DNA binding. Binding to core RNAP induces or stabilizes an “open,” active σ conformation, optimal for promoter binding (Callaci et al., 1999; Schwartz et al., 2008; Vishwakarma et al., 2018). Canonically, RNAP activity at promoters is regulated through DNA-binding transcription factors (Browning and Busby, 2016) that recognize and bind to specific motifs on dsDNA (DB-TFs) and influence the initiation pathway steps after promoter binding (Figure 1A). A number of proteins, called σ-regulators in this review, have evolved to tune the structure of the σ/core RNAP interaction, thus altering RNAP promoter selectivity and activity globally. These RNAP-binding transcription factors (RPB-TFs) bind to RNAP before the RNAP-promoter complex formation upon RNAP assembly. Consequently, RPB-TFs can influence all the ensuing steps of initiation and in some cases, also elongation and termination (Figures 1A,D). These proteins can be divided in two groups: (1) σ-activators (RbpA, Crl, GcrA, and GrgA) that target the σ2 domain and consequently its interaction with the −10 element, and (2) σ-repressors (Gp39, AsiA, P7, and Scc4) that target the σ4 domain and consequently its interaction with −35 element (Table 1). All σ-repressor, but one, are phage-encoded proteins that appropriate the host transcriptional machinery during infection (Tabib-Salazar et al., 2019).

**TABLE 1:** Properties of the RNAP-binding σ-regulators.

Name	Phylum/organism	Targeted σ	Binding site	DNA interaction	Regulated process	Mode of action	Structures/PDB code
**σ-activators**							
RbpA	*Actinobacteria/Mycobacterium tuberculosis*	σ^A^, σ^B^	σ2, β′−clamp/RNA−exit channel	Nonspecific	•Growth •Stress response •Stationary phase	σ–RNAP assembly (chaperon); Stimulates RPo formation; Stimulates promoter escape	RbpA–σ^A^-RPo/6C04, 5TW1, 5VI5; RbpA-σ^A^-RNAP/6C05
Crl	γ*-Proteobacteria/Escherichia coli*	σ^S^	σ2/β′−CT	No	•Stress response •Stationary phase	σ–RNAP assembly (chaperon); Stimulates RPo formation	Crl–ITC5/6KJ6; Crl–RPo/6OMF
GcrA	α*–Proteobacteri/Caulobacter crescentus*	σ^A^	σ2	Methylated DNA (m^6^A)	•Cell cycle	Stimulates RPo formation at methylated promoters	GcrA-σ^A^/5YIX
GrgA	*Chlamydiae/Chlamydia trachomatis*	σ^A^, σ^28^	σ2	Nonspecific	Unknown	Activates transcription initiation	–
**σ-repressors**							
Scc4 (CT663)	*Chlamydiae/Chlamydia trachomatis*	σ^A^	σ4/β-FLAP	No	• Growth • Infection	Inhibits transcription initiation at −10/−35 promoters (likely by σ4 displacement)	–
Gp39	*Deinococcus-Thermus/Thermus thermophilus* Phage P23-45	σ^A^	σ4/β-FLAP	No	•Phage transcription	Inhibits RPo formation by σ4 displacement; Stimulates elongation. Anti-terminator function	RNAP-Gp39/3WOD
AsiA	γ*-Proteobacteria*/*Escherichia coli* Phage T4	σ^70^	σ4/β-FLAP	Nonspecific	•Phage transcription	Inhibits host RPo formation by σ4 appropriation (σ4 displacement); Stimulates phage RPo formation	RPo-AsiA-MotA/6K4Y
P7	γ-Proteobacteria/*Xanthomonas oryzae* Phage Xp10	No	β′−NTD/β–FLAP/RNA–exit channel	Unknown	•Phage transcription	Inhibits RPo formation by σ4 displacement; Stimulates elongation; Anti–terminator function	P7–TEC/6J9F

## Activators Targeting the σ2 Domain

### RbpA

**FIGURE 2:**
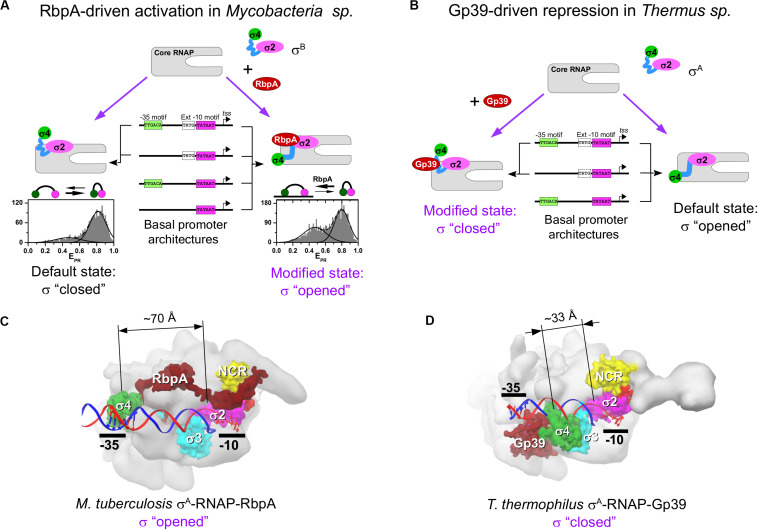
Regulation of the σ subunit conformational states by RbpA and Gp39. **(A)** Model representing the mechanism of RbpA–driven transcription activation in *Mycobacteria* sp. At the bottom, the histograms show the smFRET efficiencies (E_PR_) distributions for the double–labeled σ^B^ subunit in the RNAP holoenzyme without (left) and with RbpA (right) (data from Vishwakarma et al., 2018). **(B)** Model representing the mechanism of gp39-driven transcription repression in *T. thermophilus*. **(C)** Structure of the *Mtb*RNAP-σ^A^ RPo in complex with RbpA [Protein Data Bank (PDB) code: 6EDT]. **(D)** Structure of the *Tht* RNAP-σ^A^ RPo in complex with gp39 [Protein Data Bank (PDB) code: 3WOD]. The dimension lines show distances between Cα atoms of homologous residues in domain σ2 (*Mtb* T356, *Tht* N248) and domain σ4 (*Mtb* G497, *Tht* G391).

RbpA is a ∼14-kDa protein specific to *Actinomycetes* sp. RbpA was discovered in *Streptomyces coelicolor* as a protein that is associated with the RNAP holoenzyme (Paget et al., 2001) and is required for rapid growth and confers basal levels of rifampicin resistance (Newell et al., 2006). Later studies in *Mtb* described RbpA as a σ-specific transcriptional activator implicated in the stress response (Hu et al., 2012, 2014) and essential for growth (Forti et al., 2011). RbpA binds to group 1 and group 2 σ subunits (σ^A^ and σ^B^ in *Mtb;*σ^HrdB^ and σ^HrdA^ in *S. coelicolor*), but not to group 3 and group 4 σ subunits (Bortoluzzi et al., 2013; Tabib-Salazar et al., 2013; Hu et al., 2014). RbpA exerts multiple effects on transcription initiation. It stabilizes σ interaction with core *Mtb*RNAP, promotes DNA melting, stabilizes RPo, and accelerates promoter escape (Hu et al., 2012, 2014; Perumal et al., 2018). However, a recent study on the σ^A^-*Mtb*RNAP holoenzyme suggests that RbpA inhibits promoter escape (Jensen et al., 2019). This discrepancy indicates that RbpA effect on transcription might be promoter-specific. RbpA structure comprises an unstructured N-terminal tail (NTT), a central RbpA core domain (RCD), and a C-terminal region called the σ-interacting domain (SID) (Figure 1C). RCD and SID are connected by a flexible loop called the basic linker (BL) (Bortoluzzi et al., 2013; Tabib-Salazar et al., 2013; Hubin et al., 2015). RbpA interacts with σ2 via its SID, whereas BL (R79) interacts with promoter DNA upstream of the −10 element (Hubin et al., 2017). RbpA-SID interacts with three σ2 regions: NCR, 1.2, and 2.3 (Figure 1C). RbpA tethers σ^A^ to core RNAP via the β′-Zinc–binding domain (Hubin et al., 2015, 2017). Recent cryo-EM structures of *Mtb* RPo (Figure 2C) showed that RbpA-NTT threads through the RNA exit channel into the active site cleft and interacts with the 3.2 region of σ^A^ and the DNA template strand at position −5 (Boyaci et al., 2019). However, RbpA-SID is sufficient for partial transcription activation (Hubin et al., 2015). The complex network of interactions between RbpA and key structural modules of RNAP explains why RbpA affects different steps of initiation, from RPo formation to promoter escape. Recent single-molecule Förster resonance energy transfer (smFRET) study showed that *Mtb* σ^B^ adopts a closed, inactive conformation (∼50 Å distance between σ2 and σ4) even after assembly of the σ^B^-RNAP holoenzyme (Vishwakarma et al., 2018). During holoenzyme assembly, RbpA stabilizes (or induces) the open conformation of σ^B^ (∼83 Å distance between σ2 and σ4), required for its tight binding to core *Mtb*RNAP and to promoter DNA (Figure 2A). Thus, RbpA acts as a chaperone to promote holoenzyme formation. This finding suggests that in the absence of RbpA, part of the σ-core RNAP interface cannot be formed, thus explaining the low stability of the σ^A^ and σ^B^ *Mtb*RNAP holoenzymes (Hu et al., 2012, 2014). On the basis of the high structural similarity between σ^A^ and σ^B^ we propose that the same activation mechanism works also for σ^A^. This conclusion is supported by the cryo-EM structure of *Mycobacterium smegmatis*σ^A^-RNAP holoenzyme lacking electron density for the domain σ4. This indicates that σ fluctuates between different conformational states (Kouba et al., 2019). The smFRET study on σ^B^-*Mtb*RNAP also explains why RbpA is essential for transcription initiation at the −10/−35 promoters and dispensable at the extended −10 promoters (Hu et al., 2012, 2014; Perumal et al., 2018). Indeed, RPo formation at the −10/−35 promoters requires the distance between domains σ2 and σ4 to match the distance between the −10 and −35 elements. This condition is dispensable for RPo formation at the extended −10 promoter. Therefore, regulation of the σ conformational state by RbpA allows modulating RNAP promoter selectivity (Perumal et al., 2018).

### Crl

Crl is a ∼16-kDa protein from γ-proteobacteria, initially identified in *E. coli* as an activator of genes implicated in curli fimbriae production (Arnqvist et al., 1992). Crl binds to stationary phase σ^S^ and activates σ^S^-RNAP-mediated transcription, independently of the promoter sequence (Pratt and Silhavy, 1998; Bougdour et al., 2004). Although Crl does not bind to σ^70^ because of the steric clash with σ^70^-NCR (Cartagena et al., 2019) it can activate σ^70^-dependent transcription (Gaal et al., 2006). As observed for RbpA, Crl facilitates transcription initiation by stabilizing the σ^S^-RNAP holoenzyme and stimulating RPo formation (Banta et al., 2013; Xu et al., 2019). Two high resolution cryo-EM-based structures of Crl-σ^S^-RNAP RPo have been recently described (Cartagena et al., 2019; Xu et al., 2019). Cartagena et al., suggested that Crl stabilizes σ^S^-RNAP by tethering σ^S^ directly to RNAP though contacts with the β′-clamp-toe domain (β′–CT, residues 144–179). Based on structure and hydrogen–deuterium exchange mass spectrometry analysis of σ^S^ conformation, Xu et al. (2019) suggested that Crl acts as a chaperone that facilitates the σ^S^-RNAP holoenzyme assembly mainly by modifying σ2 conformation, but not through its contacts with the β′-clamp (Figure 1C) stabilizes its optimal conformation for binding to the −10 element ssDNA. This interaction promotes RPo formation. It is not known whether Crl plays any role in promoter escape. However, the finding that the β′CT/σ^70^-NCR interaction antagonizes the σ2/β′ clamp interaction and facilitaes promoter escape Leibman and Hochschild, 2007) suggests this possibility (Banta et al., 2013; Cartagena et al., 2019).

### GcrA

GcrA (173–aa) is a transcription factor from *Caulobacter crescentus* that is well conserved in α–proteobacteria. GcrA forms a stable complex with σ^A^-RNAP, recruits RNAP to methylated (m^6^A) promoters, and activates the expression of ∼200 genes that play an important role in cell cycle regulation during swarmer-to-stalked cell transition (Holtzendorff et al., 2004; Haakonsen et al., 2015). Analysis of the promoter binding kinetics demonstrated that GcrA increases RNAP affinity for the promoter and the rate of RPc isomerization to RPo (Haakonsen et al., 2015). GcrA is composed of two domains: the N-terminal DNA-binding domain (GcrA-DBD, residues 1–45) that recognizes methylated promoter DNA, and the C-terminal σ-interacting domain (GcrA-SID, residues 108–173) that binds to σ2 (Figure 1C). GcrA-DBD and GcrA-SID are connected by an unstructured linker (residues 46–107). Recent crystal structures of the GcrA-SID-σ^A^ complex and the GcrA-DBD-DNA complex revealed details of its interactions with RNAP and the promoter (Wu et al., 2018). Structural studies on the full length protein and its complex with RNAP are now needed to decipher GcrA mechanism of action.

### GrgA

GrgA (ORF CTL0766, 288-aa) is a transcription factor from the human pathogen *Chlamydia trachomatis*. GrgA activates σ^A^- (also known as σ^66^) and σ^28^-dependent transcription by interacting with σ-NCR and binding to DNA in a non-sequence-specific manner (Bao et al., 2012; Desai et al., 2018). The GrgA binding site on σ^A^ was mapped to residues 269–316 (Bao et al., 2012) (Figure 1C). The detailed mechanism of GrgA action and its role in gene regulation remain obscure. GrgA is specific to *Chlamydia* species, and has not been found in any other organism. Therefore, it might be a good target for developing highly selective anti-chlamydial drugs (Zhang et al., 2019).

## Repressors Targeting the σ4 Domain

### Gp39 of Phage P23-45

Gp39, a ∼16-kDa protein encoded by the *Thermus thermophilus* phage P23-45, binds to the host σ^A^-RNAP holoenzyme and inhibits transcription from −10/−35 class promoters. Transcription of the middle and late promoters of P23-45, which belong to the extended −10 class, is less affected (Berdygulova et al., 2011; Tagami et al., 2014). Gp39 blocks transcription initiation probably at the step of RPc formation that depends on the σ4/−35 element contact. Besides its effect on initiation, gp39 also displays anti-termination activity (Berdygulova et al., 2012) suggesting that the σ subunit is not essential for its binding to RNAP. The crystal structure of the σ^A^-RNAP-gp39 complex (Tagami et al., 2014) reveled that gp39 binds to the RNAP β-flap and to the σ4 domain and induces a ∼45 Å displacement of the σ4 relative to its default position in the RNAP holoenzyme (Figures 2B,D). This conformational change in the σ^A^ subunit explains the selectivity of the RNAP-gp39 complex toward the extended −10 promoters.

### AsiA of Phage T4

AsiA is 90-aa protein of the *E. coli* phage T4. AsiA employs a mechanism called σ appropriation to reprogram the host RNAP. AsiA forms a stable complex with σ^70^ before holoenzyme assembly (Hinton et al., 1996; Hinton and Vuthoori, 2000) and thus inhibits transcription from the −10/−35 class promoters. Conversely, transcription from the extended −10 promoters is less affected (Severinovaa et al., 1998). At the same time, AsiA acts as a co-activator of the phage activator protein MotA, required for binding to the T4 middle promoter. The σ appropriation complex, which includes σ^70^, RNAP, AsiA and MotA, recognizes the MotA-box that replaces the −35 element at the T4 middle promoters. NMR solution structures of the AsiA-σ4 complex demonstrated that AsiA remodels σ4 making impossible its binding to the −35 element and its interaction with β-flap (Simeonov et al., 2003; Lambert et al., 2004). A recent cryo-EM structure of the σ^70^-RNAP-AsiA-MotA RPo revealed the detailed mechanism of σ appropriation (Shi et al., 2019). AsiA binds to and remodels the structure of the σ region 3.2 and σ4, displaces σ4, and takes its place. This allows MotA recruitment and RPo formation. In addition, AsiA interaction with upstream dsDNA stabilizes RPo.

### P7 of Phage Xp10

P7 is a small, ∼ 8-kDa, globular protein encoded by the lytic bacteriophage Xp10 that infects the Gram-negative bacterium *Xanthomonas oryzae*, which causes rice blight. At a later stage of infection, P7 shuts off the host gene transcription in favor of phage gene transcription by the Xp10 single-subunit RNAP (Nechaev et al., 2002; Liu et al., 2014). P7 forms a stable complex with the host σ^70^-RNAP holoenzyme and inhibits RPo formation at −10/−35 promoters and to a lesser extent, at the extended −10 promoters. Luminescence resonance energy transfer (LRET) measurements demonstrated that in the P7-σ^70^-RNAP complex, the σ^70^ subunit adopts a closed or partially closed conformation (Nechaev et al., 2002). The finding that P7 also binds to RNAP harboring the structurally distinct σ^54^ (Brown et al., 2016) suggests that the σ subunit is not essential for its interaction with RNAP. Indeed, P7 can also modulate post-initiation steps of transcription, such as pausing and intrinsic termination (Nechaev et al., 2002; [Bibr B64][Bibr B62]). A recently solved cryo-EM structure of P7 in the elongation complex (You et al., 2019) reveled that P7 binds to the RNA-exit channel at the place of σ4, and thus makes impossible the formation of the “open” σ conformation essential for RPo formation at −10/−35 class promoters. The lack of σ4-RNAP contact should decrease the overall stability of the holoenzyme, thus explaining the dissociation of σ from the P7-RNAP complex observed in biochemical experiments (Liu et al., 2014). It has been proposed that P7 induces the closed conformation of the RNAP clamp, and thus inhibits RPo formation (You et al., 2019). However, it is unlikely that such mechanism takes place at −10/−35 promoters. Indeed, according to the P7-RNAP complex structure, P7 should inhibit the interaction of σ4 with the −35 element, which is required for initial RNAP binding to the promoter (RPc formation). Clamp closing starts to play a role during RPc isomerization to RPo, the step following recognition of the −35 element. Thus, it is more likely that P7-mediated σ^70^ remodeling inhibits the σ4/−35 element interaction and consequently RPc formation, as observed for the σ^54^-RNAP holoenzyme (Brown et al., 2016). However, P7-induced clamp closing might play a role when the −35 element recognition is bypassed.

### Scc4 (CT663) From *Chlamydia trachomatis*

Scc4 (ORF CT663) is a ∼15-kDa protein from the human pathogen *C. trachomatis.* Scc4 forms a heterodimer with Scc1, and both are type III secretion chaperons implicated in the regulation of cell growth and intracellular infection (Hanson et al., 2015). Scc4 was identified in a two-hybrid screen for regulators that interact with *C. trachomatis* RNAP β-FLAP (Rao et al., 2009). Scc4 binds to RNAP β-FLAP tip helix and also interacts with the σ4 domain of the principal σ^A^ subunit. It can also interact with the σ4 domain of *E. coli* σ^70^ that exhibits 60% amino acid identity with the σ4 of σ^A^, but does not interact with the σ4 of *C. trachomatis* σ^28^ (Group 3). Scc4 inhibits transcription initiation from −10/−35 class promoters, but not from extended −10 type promoters. Although structural studies are needed to determine the mechanism of inhibition, on the basis of similarities with the mechanism of action of the phage proteins we hypothesize that Scc4 disrupts the σ4/Flap interaction and prevents RPo formation at −10/−35 promoters.

## Conclusion

We can draw two basic principles of transcription regulation by RPB-TFs: positive regulation through strengthening of σ2/β′10 element interactions, and negative regulation through weakening of σ4/β-flap/−35 element interactions. All contacts made by the three σ-activators RbpA, Crl and GcrA overlap and are clustered in four σ regions (σ1.2, σ-NCR, σ2.1 and σ2.3) that are responsible for core RNAP binding and −10 element recognition/melting (Figure 1C). Consequently, all these activators act through a similar mechanism. They strengthen σ/RNAP interaction and stimulate RPo formation, the rate limiting step in transcription initiation. The only exception is GrgA the binding site of which was mapped entirely to σ NCR and thus may have a different mechanism of action. However, in the absence of a detailed biochemical and structural characterization, it cannot be excluded that GrgA contacts other regions besides σ-NCR.

At least for RbpA, the stimulation of the “closed-to-open” transition is part of the σ activation mechanism required for efficient transcription initiation at the −10/−35 class promoters, but not at the extended −10 class promoters (Vishwakarma et al., 2018). It remains to be explored whether Crl, GcrA and GrgA can affect the relative movement of the σ2 and σ4 domains. Remarkably, all σ-repressors mentioned here act as antagonists to RbpA-type activation by destabilizing the σ4/β-flap interaction, and should favor the “open-to-closed” transition in the σ subunit. Consequently, σ-repressor-modified RNAP cannot initiate transcription at the −10/−35 class promoters, but only at the extended −10 class promoters (Figure 2A,B).

The σ-activators and σ-repressors illustrate how σ conformational dynamics, controlled by contacts with core RNAP, can be used for fine-tuning transcription in a lineage-specific manner. Considering the huge diversity in lifestyles of bacterial species, the number of the currently known σ-regulators of bacterial origin is strikingly low. The reason might be that most of these proteins are of small size and are not easy to detect. Yet, their discovery in pathogenic bacteria may offer new targets for developing pathogen-specific drugs. We expect that the number of the described σ-regulators and the diversity of regulatory mechanisms will continue to grow.

## Author Contributions

KB and RV wrote the manuscript. Both authors contributed to the article and approved the submitted version.

## Conflict of Interest

The authors declare that the research was conducted in the absence of any commercial or financial relationships that could be construed as a potential conflict of interest.
